# Assessing the Prevalence of Self-Reported Backache and Neck Pain and Their Contributing Risk Factors Among Medical Students: A Cross-Sectional Study

**DOI:** 10.7759/cureus.99091

**Published:** 2025-12-13

**Authors:** Asmaa A Altarqi, Sumayyah A Albayti, Reem I Albawab, Sara A Alharthi, Alredaa H Farran, Shaimaa R Abdelmohsen

**Affiliations:** 1 Medicine and Surgery, Ibn Sina National College, Jeddah, SAU; 2 Anatomy, Ibn Sina National College, Jeddah, SAU

**Keywords:** backache, jeddah, medical students, risk factors, stress

## Abstract

Background

Backache is a common concern, particularly within academic environments, and is one of the most frequent reasons for medical consultations after upper respiratory tract infections. Medical students, in particular, represent a demographic group that is highly susceptible to developing backache due to their unique lifestyle and academic demands. As a prevalent musculoskeletal complaint worldwide, backache significantly impacts both health and quality of life, affecting a wide range of individuals, including young adults such as university students.

Aim

This study aimed to assess the prevalence of backache and investigate its statistical association with demographic factors, stress, and study posture among medical students.

Methodology

A cross-sectional study was conducted with 452 undergraduate medical students from Ibn Sina Medical College, Jeddah, Saudi Arabia. A convenience sampling technique was used to select students who reported backache. Data were collected through structured online questionnaires, covering demographic information; presence and location of back pain; onset; medical consultations; study positions; pain relief methods; relationship with stress; and duration of backache.

Results

The majority of the participants, 396 (87.6%), were females, of whom 348 (85%) reported experiencing backache, with 195 (50%) specifically reporting neck pain. Among those with backache, 148 (38%) studied in an office setting. No statistically significant correlation was found between backache and study positions (p = 0.125). However, a Chi-square analysis indicated a significant association between backache and being a female medical student (p = 0.001) or experiencing stress (p = 0.001).

Conclusion

This study emphasizes the high prevalence of backache among medical students and underscores the need for educational institutions and healthcare providers to address the associated factors. Further medical research can help create a healthier academic environment, as well as promote the well-being of future medical professionals.

## Introduction

Backaches are a significant health concern among medical students, who are particularly vulnerable due to the rigorous demands of their education and training. This population faces a unique combination of stressors, including prolonged study hours, sedentary postures, and psychological stress, all of which contribute to musculoskeletal issues [1]. Backaches are among the most prevalent reasons individuals seek emergency care and are commonly attributed to poor posture, with mechanical or non-specific causes being the most frequent [1,2].

Backaches can manifest as acute, subacute, or chronic conditions, with acute back pain being the most common and typically self-limiting [3]. Among medical students, who often engage in prolonged periods of study, back pain is particularly prevalent. For example, a study at Qassim University found a high prevalence of neck pain (60.8%), lower back pain (LBP) (46.8%), and shoulder pain (40.0%) among students [4,5].

Several factors contribute to back pain in this demographic. These include prolonged sitting, poor ergonomic practices, stress, and anxiety. Gender differences have also been observed, with a higher prevalence of back pain reported among female students [6-8]. In Saudi Arabia, studies have reported a high prevalence of LBP among medical students, including a 67% prevalence over 12 months at central Saudi universities, 50.1% over the past week at Umm Al-Qura University, and 35.5% among male students at Taif University [9,10].

Despite these findings, there is limited research focusing on the prevalence and associated risk factors of backaches among medical students in Jeddah, Saudi Arabia. This study aims to address this gap by examining the prevalence and associated factors of back pain in this population, specifically among medical students aged 18-30. The primary objective of this study was to estimate the prevalence and characteristics (location, duration, and onset) of backache among medical students in Jeddah. The secondary objective was to examine the relationship between backache and potential contributing factors, specifically gender, perceived stress levels, and study positions, using inferential statistical analysis. By identifying these associations, this study aims to provide data that can support future preventive strategies in academic settings.

By investigating these issues, this study seeks to provide a better understanding of back pain among medical students in Jeddah and to encourage preventive measures that can foster a healthier academic environment.

## Materials and methods

Study design

A cross-sectional analytic study was conducted from December 2022 to March 2023 to investigate the prevalence, features of backaches, and contributing risk factors among medical students.

Study context

The study took place at a medical college with four programs: Medicine, Dentistry, Pharm D, and Nursing. Students share a common foundation year before progressing into their specific fields, so this research focused on medical students in Years 2 to 6. Excluded from the study were students or interns from outside Ibn Sina National College, Jeddah, Saudi Arabia; those with trauma-related pain, rheumatological diseases, or knee and spinal diseases; and participants with incomplete responses. The study used a convenience sampling method, including only those who were readily available and agreed to participate.

Sample size

The G*Power Software (Heinrich-Heine-Universität Düsseldorf, Düsseldorf, Germany) [11] determined the minimum required sample size to be 220 participants, with α = 0.05, β = 0.95, and a degree of freedom of 5. All Years 2-6 students in the Medicine Program (approximately 1,500 students) were invited to participate. Out of this eligible population, 452 complete responses were received, yielding a response rate of approximately 30.1%. This sample size significantly exceeded the minimum requirement calculated by the power analysis.

Data collection

A structured online questionnaire (see Appendix 1), available in both Arabic and English, was distributed via official institutional communication channels, including class WhatsApp groups and university email lists for students in Years 2 through 6. To minimize selection bias, the survey was posted at different times of the day over a 12-week period. The survey platform (Google Forms; Google, Inc., Mountain View, CA, USA) was configured to prevent multiple submissions from the same user. The questionnaire, developed based on an extensive literature review and expert consultation, assessed the prevalence of backaches, risk factors, and their impact on daily activities. The survey was designed for completion within three to four minutes.

Data analysis

Data were encoded and analyzed using IBM SPSS Statistics for Windows, Version 25 (Released 2017; IBM Corp., Armonk, NY, USA). Prior to analysis, data cleaning was performed; surveys with incomplete responses were excluded to ensure dataset integrity (listwise deletion).

Descriptive statistics, including frequencies and percentages, were used to summarize categorical demographic data (gender and academic year) and prevalence features (site of pain and duration). Means and standard deviations (SD) were calculated for continuous variables (age).

Inferential statistical tests were applied to generate the p-values regarding risk factors: (i) Pearson’s Chi-square test: This was used to determine the statistical significance of associations between categorical independent variables, specifically gender, study position (office vs. non-office), and perceived stress, and the dependent variable (presence of backache). (ii) Independent Samples t-test: This was conducted to compare the continuous variable (age) between the two groups (students with backache vs. students without backache) to determine if age was a significant differentiating factor.

All statistical tests were two-tailed, and a p-value of less than 0.05 (p < 0.05) was considered statistically significant.

Ethical considerations

In compliance with the Helsinki Declaration [12], ethical permission was acquired from the college's Institutional Research Review Board (REF No. 029MP/CR28032023). The right to decline participation in the study for any reason, without consequence, was granted to the students. Information was kept confidential, and the online survey was distributed in an anonymous manner.

## Results

Participant demographic characteristics

Data for this study were collected from a total of 452 male and female medical students. Reliability coefficients (Cronbach’s alpha) were calculated for various age groups and genders. The majority of participants were in the age group of 21-33 years (199, 44%), followed by those aged 17-20 years (126, 27.9%). Most participants identified as female (396, 87.6%), while 56 (12.4%) identified as male (Table 1).

**Table 1:** Demographic characteristics of the study participants with respect to age and gender This table provides a detailed breakdown of the study participants by age and gender, offering insights into the demographic composition of the sample.

		Count	Percentage
Age	17-20 years	126	27.9
	21-23 years	199	44
	24-26 years	74	16.4
	27-30 years	53	11.7
Gender	Female	396	87.6
	Male	56	12.4

Prevalence and features of backaches

A significant number of participants reported having backache (384, 85%), while only a small percentage did not suffer from backaches (68, 15%). Half of them reported neck pain (195, 50%), while the other half experienced other types, including upper back pain (80, 23.2%), LBP (lumbar pain) (50, 13%), and shoulder pain (15, 3.9%). Regarding the duration of pain, 131 (34%) of the students had been suffering from back pain for months, 128 (33.3%) for years, and the rest for days or a few weeks. A notable percentage reported sudden onset of pain (182, 47.4%). When asked about their study posture, most students studied sitting in the office (148, 38%), 123 (32%) lying on their beds, while the rest studied on the couch (64, 16.7%), on the floor (34, 8.9%), walking (3, 0.8%), or standing (2, 0.5%). More than half of the students agreed that the pain increased with stress (234, 60.9%) (Table 2).

**Table 2:** Prevalence of the backaches and associated features This table summarizes the prevalence of backaches among students, detailing the sites of pain, duration, onset, and study positions.

Variable	Categories	Number (N)	Percentage
Backaches	Yes	384	85.0
	No	68	15.0
Site of pain	Upper pain	325	87
	Lower pain	50	13
Duration of the backaches	Days	61	15.9
	Weeks	64	16.7
	Months	131	34.1
	Years	128	33.3
Onset of pain	Suddenly	182	47.4
	Gradually	80	20.8
	With special position	109	28.4
	After trauma	13	3.4
Position of the study	Office	148	38
	Non-office	236	61.5

Comparative analysis of risk factors for back pain 

A comparative analysis was conducted to examine the relationship between having a backache and the site of study. There was an insignificant difference in the incidence of back pain between students who sit at a desk (office) and those who sit on the floor (p = 0.125). There was a significant difference in the incidence of back pain during stress compared with students without stress (p = 0.001). The results also showed that being female is associated with a higher incidence of backaches than being male (p = 0.001) (Table 3).

**Table 3:** Correlation between backaches and risk factors The table shows significant correlations between backaches and gender and stress, and an insignificant correlation between backaches and the site of study.

Backache	Studying in in the office	Studying on the floor	p-value
Yes	148 (88.1%)	34 (79.1%)	0.125
No	20 (11.9%)	9 (20.9%)	
	Related to stress	Not related to stress	
Yes	234 (60.9%)	25 (36.8%)	0.001
No	150 (39.1%)	43 (63.2%)	
	Female	Male	
Yes	347 (90.4%)	37 (9.6%)	0.001
No	49 (72.1%)	19 (27.9%)	

Relationship with baseline features: quantitative statistical analysis

Table 4 shows an insignificant difference between age and the presence of backaches (p = 0.808, t = 0.244).

**Table 4:** Relation between backaches and baseline characteristics There was an insignificant difference in the age between students who have backaches and whose haven’t while using the office during studying.

Backaches	Age		t-test	p-value
	Mean	Standard deviation		
Yes	22.56	± 3.33	0.244	0.808
No	22.75	± 2.63		

## Discussion

Backaches represent a substantial and growing health concern globally, contributing more to disability than any other condition [10]. It is estimated that up to 80% of individuals will experience low back pain at some point in their lives [13]. Early onset of backaches can often continue into adulthood, highlighting the importance of prevention and effective management strategies for younger populations to prevent chronicity [14].

University students, particularly medical students, face a high risk of developing backaches due to extended sedentary study hours and significant psychosocial stressors. Previous studies have reported prevalence rates of backaches among university students ranging from 25% to 66.4% [15,16], with medical students frequently at the higher end of this spectrum, due to their intense cognitive demands and mental strain [17]. Our study reveals an exceptionally high prevalence of backaches, with 384 (85%) of participating medical students at Ibn Sina National College reporting symptoms. This is notably higher than the 50%-70% prevalence reported in similar cohorts [18,19], underscoring the severe impact of back pain on this population.

A total of 384 (50%) of participants in our research study suffered from back pain, with neck pain being the most common, impacting 50% of students. This is in line with earlier studies, which have shown that student neck pain often results from working at a table or desk for long hours, in combination with not using an appropriate posture [5,6]. Medical students are especially vulnerable, given their extended study hours and the increased use of digital devices, which often lead to forward head posture, a known contributor to neck pain [20].

Concerns of chronicity abound, as up to one-third of students in our study reported neck or back pain experienced for many months to years. It seems that the acute pain experienced by many students, due to poor posture coupled with prolonged sitting, can become chronic without appropriate intervention [21]. Neck pain is a key player in the initial appearance of musculoskeletal strain in academic settings. At this point, early intervention, involving ergonomic training and posture correction, might be able to halt that progression.

Interestingly, there was no relationship between study posture and complaints of backache, including neck pain (p = 0.125). This is contrary to what some studies have suggested, which state that low study environments and poor posture are risk factors for back pain [21]. Other factors, such as total sitting time and stress level, might also be stronger predictors of pain development, despite students reporting that they studied more often at their desks. Better prevention outcomes might result from addressing such factors, particularly in connection with neck pain, rather than focusing on posture alone.

A notable finding is the strong association between stress and back pain (p = 0.001), especially when the neck is the site of pain (p = 0.006). Stress is associated with increased muscle tension, particularly in the neck and shoulder area, which enhances pain and thus promotes slower recovery from injuries [22]. The strong association of stress with neck pain indicates the importance of multi-modal approaches to care, including interventions beyond traditional physical ones.

In addition, the present study reported that more female students suffered from backaches, with a significant increase in neck pain compared to their male counterparts (p = 0.001). It is important to note that females constituted 87.6% of our study sample. While this reflects the demographic distribution of respondents in this convenience sample, the statistical association remains significant within the group. This is consistent with previous literature, which has shown that women are at an increased risk of musculoskeletal disorders, possibly due to differences in anatomy, hormonal changes, as well as greater psychosocial stress [23]. Tailoring interventions to account for these gender differences may be critical in effectively managing neck and back pain in female students.

Limitations

This study has several limitations that must be considered when interpreting the results. First, the cross-sectional design restricts the ability to establish causality between risk factors (such as stress or study position) and backache. Second, selection bias is present due to the convenience sampling method and the demographic skew toward female participants (87.6%), which limits the generalizability of the findings to male medical students. Third, the reliance on self-reported data introduces recall bias - particularly regarding the duration and exact onset of pain - and measurement bias, as pain intensity was subjective and not validated by physical examination or radiological imaging. Fourth, while the study assessed stress and posture, it did not account for potential confounding variables such as sleep quality, mattress type, specific hours of physical exercise, or pre-existing psychological conditions, which could influence musculoskeletal health. Finally, the study was designed as a descriptive epidemiological assessment and did not utilize a specific theoretical framework (e.g., the Biopsychosocial Model) to structure the variable selection, which limits the theoretical depth of the observed associations.

Recommendations

Future research should focus on identifying predictors of chronic back pain to guide early intervention strategies. Longitudinal studies are needed to track the development of back pain and the effectiveness of various interventions. Additionally, research should explore the role of psychological factors in back pain and evaluate the efficacy of different therapies among medical students.

## Conclusions

This study highlights that backaches, specifically neck pain, are a pervasive issue affecting the majority of medical students at Ibn Sina Medical College, often resulting in long-term discomfort. The significant impact of stress on back pain and the observed gender-related disparities underscore the need for comprehensive management strategies. Surprisingly, study posture did not show a relationship with back pain, suggesting that ergonomic modifications alone may be insufficient. It is crucial to address stress management and consider gender-specific factors alongside conventional physiotherapeutic approaches.

## Appendices

Appendix 1

**Table 5:** Questionnaire used in the study

Question	Response options
Gender	Female, Male
Age	18-25, 26-30, 31-35, More than 35
Program	MBBS, BDS, Pharma, Nurse
If MBBS which year	Foundation, 2nd, 3rd, 4th, 5th, 6th
Do you have backaches?	Yes, No
Location of pain	Neck pain, Upper back pain, Lower back pain, Shoulder pain, Radiating pain
Duration of backaches	Days, Weeks, Months, Years
Onset of backache	Gradually, Suddenly, After trauma
Have you visited a doctor?	Yes, No
Where are you staying?	Office, Floor, Bed, Couch, Other (please specify)
What relieves your backaches?	Painkiller, Exercise (Stretching), Sleeping, Change position, Other (please specify)
Does stress increase backaches?	Yes, No

## References

[1] Asad G, Alam MM, Akhtar MW (2024). Prevalence of low back pain in medical students due to prolonged sitting. J Health Rehabil Res.

[2] Oertel J, Sharif S, Zygourakis C, Sippl C (2024). Acute low back pain: epidemiology, etiology, and prevention: WFNS spine committee recommendations. World Neurosurg.

[3] Chou R, Qaseem A, Snow V, Casey D, Cross JT Jr, Shekelle P, Owens DK (2007). Diagnosis and treatment of low back pain: a joint clinical practice guideline from the American College of Physicians and the American Pain Society. Ann Intern Med.

[4] Jahre H, Grotle M, Smedbråten K, Dunn KM, Øiestad BE (2020). Risk factors for non-specific neck pain in young adults: a systematic review. BMC Musculoskelet Disord.

[5] Alsalameh AM, Harisi MJ, Alduayji MA, Almutham AA, Mahmood FM (2019). Evaluating the relationship between smartphone addiction/overuse and musculoskeletal pain among medical students at Qassim University. J Family Med Prim Care.

[6] Vujcic I, Stojilovic N, Dubljanin E, Ladjevic N, Ladjevic I, Sipetic-Grujicic S (2018). Low back pain among medical students in Belgrade (Serbia): a cross-sectional study. Pain Res Manag.

[7] Aggarwal N, Anand T, Kishore J, Ingle GK (2013). Low back pain and associated risk factors among undergraduate students of a medical college in Delhi. Educ Health (Abingdon).

[8] Alturkistani LH, Hendi OM, Bajaber AS, Alhamoud MA, Althobaiti SS, Alharthi TA, Atallah AA (2020). Prevalence of lower back pain and its relation to stress among medical students in Taif University, Saudi Arabia. Int J Prev Med.

[9] Algarni AD, Al-Saran Y, Al-Moawi A, Bin Dous A, Al-Ahaideb A, Kachanathu SJ (2017). The prevalence of and factors associated with neck, shoulder, and low-back pains among medical students at University Hospitals in central Saudi Arabia. Pain Res Treat.

[10] Hartvigsen J, Hancock MJ, Kongsted A (2018). What low back pain is and why we need to pay attention. Lancet.

[11] Burton AK, Clarke RD, McClune TD, Tillotson KM (1996). The natural history of low back pain in adolescents. Spine (Phila Pa 1976).

[12] Muyor JM (2013). Exercise intensity and validity of the ratings of perceived exertion (Borg and OMNI scales) in an indoor cycling session. J Hum Kinet.

[13] Balagué F, Mannion F, Pellisé F (2012). Non-specific low back pain - authors’ reply. Lancet.

[14] Coenen P, Smith A, Paananen M (2017). Trajectories of low back pain from adolescence to young adulthood: the Raine study. Arthritis Care Res (Hoboken).

[15] Tavares C, Salvi CS, Nisihara R, Skare T (2019). Low back pain in Brazilian medical students: a cross-sectional study. Int J Rheum Dis.

[16] Alshehri MM, Alqhtani AM, Gharawi SH (2023). Prevalence of lower back pain and its associations with lifestyle behaviors among college students in Saudi Arabia. BMC Musculoskelet Disord.

[17] Alshagga MA, Nimer AR, Yan LP, Ibrahim IA, Al-Ghamdi SS, Radman Al-Dubai SA (2013). Prevalence and factors associated with neck, shoulder and low back pains among medical students in a Malaysian Medical College. BMC Res Notes.

[18] Taha YA, Al Swaidan HA, Alyami HS (2023). The prevalence of low back pain among medical students: a cross-sectional study from Saudi Arabia. Cureus.

[19] Amyra Natasha A, Ahmad Syukri A, Siti Nor Diana MK, Ima-Nirwana S, Chin KY (2018). The association between backpack use and low back pain among pre-university students: a pilot study. J Taibah Univ Med Sci.

[20] Kim MH, Yi CH, Kwon OY, Cho SH, Yoo WG (2008). Changes in neck muscle electromyography and forward head posture of children when carrying schoolbags. Ergonomics.

[21] Geldhof E, Cardon G, De Bourdeaudhuij I, Danneels L, Coorevits P, Vanderstraeten G, De Clercq D (2007). Effects of back posture education on elementary schoolchildren's back function. Eur Spine J.

[22] Pinheiro MB, Ferreira ML, Refshauge K (2015). Symptoms of depression predict new episodes of low back pain: A systematic review and meta-analysis. Arthritis Care Res (Hoboken).

[23] Wijnhoven HA, de Vet HC, Smit HA, Picavet HS (2006). Hormonal and reproductive factors are associated with chronic low back pain and chronic upper extremity pain in women--the MORGEN study. Spine (Phila Pa 1976).

